# Excess Mortality and Causes Associated with Chikungunya, Puerto Rico, 2014–2015

**DOI:** 10.3201/eid2412.170639

**Published:** 2018-12

**Authors:** André Ricardo Ribas Freitas, Maria Rita Donalisio, Pedro María Alarcón-Elbal

**Affiliations:** Secretaria de Saúde de Campinas, Campinas, Brazil (A.R.R. Freitas);; Faculdade de Medicina São Leopoldo Mandic, Instituto de Pesquisas São Leopoldo Mandic, Campinas (A.R.R. Freitas);; Universidade Estadual de Campinas, Campinas (M.R. Donalisio);; Universidad Iberoamericana, Instituto de Medicina Tropical & Salud Global, Santo Domingo, Dominican Republic (P.M. Alarcón-Elbal)

**Keywords:** mortality, arboviruses, chikungunya virus, epidemiology, viruses, vector-borne infections, Puerto Rico, United States

## Abstract

During 2014–2015, a total of 31 deaths were associated with the first chikungunya epidemic in Puerto Rico. We analyzed excess mortality from various causes for the same months during the previous 4 years and detected 1,310 deaths possibly attributable to chikungunya. Our findings raise important questions about increased mortality rates associated with chikungunya.

In December 2013, the first locally acquired chikungunya virus infections in the Americas were reported in Saint Martin. Since that report, the virus has spread to 45 countries and territories in North, Central, and South America, causing ≈2.4 million suspected and confirmed cases and 440 deaths through December 2016 (*1*).

Chikungunya has emerged worldwide since 2004. Several gaps in knowledge exist about the disease and its consequences. Until recently chikungunya was considered a nonlethal disease, but severe forms and deaths have been described since an epidemic on Réunion Island during 2005–2006 (*2*).

In Puerto Rico, the chikungunya epidemic began in May 2014 as the first occurrence of the virus in the country (*3*). Official surveillance reported 28,327 suspected chikungunya cases, of which 4,339 were laboratory-confirmed; 31 persons died (0.9 deaths/100,000 population). The chikungunya mortality rate was significantly lower than that observed in epidemics on other islands, such as Réunion Island (25.9 deaths/100,000 population in 2006), Martinique (20.5/100,000 population in 2014), and Guadaloupe (14.4/100,000 population in 2014) (*1*,*4*). These differences could be a consequence of the difficulty of recognizing the etiology of severe clinical forms and deaths.

## The Study

**Figure 1:**
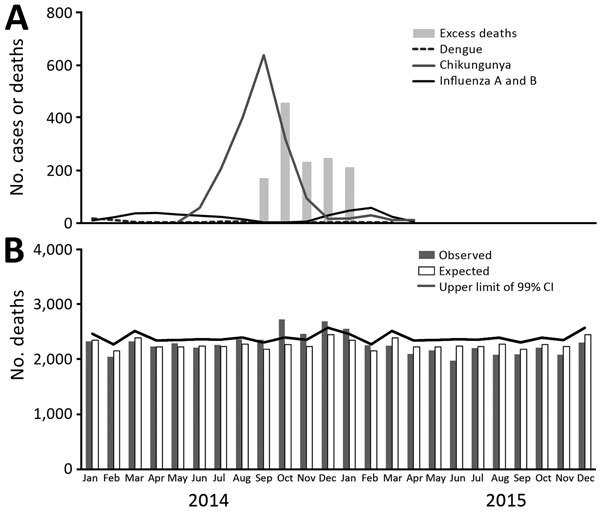
Expected and observed monthly deaths in Puerto Rico, 2014–2015. A) Excess number of monthly deaths and number of chikungunya, dengue, and influenza A and B cases diagnosed among patients with acute febrile illness in St. Luke’s Episcopal Hospital, Puerto Rico, January 2014–December 2015. Data for acute febrile illness were discontinued in April 2015. B) Number of monthly deaths, observed deaths, and expected deaths and upper limit of 99% CI, Puerto Rico, January 2014–December 2015.

We studied excess mortality associated with chikungunya in Puerto Rico by comparing monthly expected deaths and actual deaths during the epidemic (*5*–*7*). We calculated expected deaths by the average age-adjusted mortality rate for each month for 2010 to 2013 and projected them to the estimated population for 2014 and 2015 (*6*). We considered the difference between observed and expected deaths for the months in which observed deaths exceeded the upper limit of the 99% CI (*6*) as excess deaths associated with the chikungunya epidemic (Figure 1).

We estimated the mortality rate expected for the second half of 2014, the worst period of the epidemic, and compared it with the observed mortality rate. This second group of calculations was based on the all-cause age-specific mortality rate for the 10 leading causes of death in Puerto Rico and main causes of deaths associated with severe chikungunya in Réunion Island (*2*).

We obtained data on estimated population from the US Census Bureau (*8*) and on deaths from the Centers for Disease Control and Prevention’s National Center for Health Statistics (*9*). In mid-September 2014, Puerto Rico issued an official administrative order stating that only persons hospitalized with suspected chikungunya should be reported to health authorities in Puerto Rico (*3*), Thus, we monitored the occurrence of chikungunya, dengue, and other viral diseases in Puerto Rico using secondary data from published studies evaluating the etiology of acute febrile illness of any patient, such as sentinel surveillance at St. Luke’s Episcopal Hospital in the cities of Ponce and Guayama (*10*,*11*).

**Figure 2:**
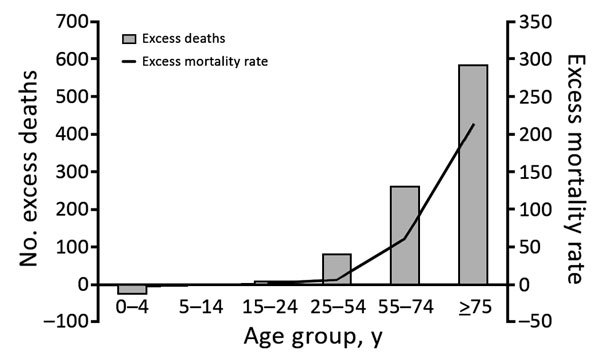
Excess deaths and difference between observed and expected deaths during chikungunya epidemic, Puerto Rico, July–December 2014. Mortality rate is deaths per 100,000 population.

We determined an excess of 1,310 deaths concurrent with the peak of the chikungunya epidemic of 2014 in Puerto Rico. We found no substantial occurrence of dengue, influenza, or leptospirosis during the chikungunya epidemic, according to data from St. Luke’s Episcopal Hospital (Figure 1) and official Puerto Rico surveillance (*10*,*12*), reinforcing the possibility that chikungunya might be etiologically associated with the deaths. Although the most affected age group was persons >75 years of age, a statistically significant proportion of deaths occurred in persons 24–55 years of age, suggesting chikungunya-associated deaths are not exclusive to elderly persons (Figure 2).

The estimated mortality rate was 30.1 deaths/100,000 population, compatible with the excess deaths observed in Réunion Island in 2006 (33.8/100,000 population) using similar methods (*5*) and slightly below the estimated rate of 47.9 deaths/100,000 population in Pernambuco, the most affected state of Brazil, during the chikungunya epidemic of 2016 (*7*). We estimated excess deaths as 42 times greater than the 31 deaths identified by the official surveillance developed during the chikungunya epidemic in Puerto Rico (*3*). In a similar study conducted in northeastern Brazil, we found 7,231 excess deaths in the region during the chikungunya epidemic of 2016, when the official surveillance system confirmed only 120 deaths, 60 times lower (*7*).

We detected a 1-month lag between the peak of chikungunya identified by St. Luke’s Episcopal Hospital and the peak of excess deaths. Other lags also were observed in similar studies in Ahmedabad, India; Mauritius; and recently in Brazil (*6*,*7*); these lags can be explained by prolonged hospitalization before death (*2*).

Deaths classified as “all other forms of chronic ischemic heart disease” (International Classification of Diseases, 10th Revision, codes I20, I25.1–I25.9) and diabetes mellitus (E10–E14) increased significantly, but deaths associated with other important causes of death, such as acute myocardial infarction (I21, I22) and neoplasms (C00–D49), did not (Table 1). These findings suggest that chikungunya contributes substantially to severity through its own pathophysiologic mechanisms and that some preexisting conditions participate only as additional risk factors for death. The most frequent clinically worsening conditions described in laboratory-confirmed chikungunya in other settings were heart failure, multiple organ failure, acute hepatitis, encephalitis, epidermolysis bullosa, myocarditis, respiratory failure, and renal failure (*2*). Many of these causes of death on Réunion Island (*3*) also increased over previous years in Puerto Rico during the epidemic (Table 2). In this study, volume depletion, emphysema, arrhythmias, asthma, diabetes mellitus and chronic ischemic heart disease led to a higher mortality rate during the epidemic than during previous years; all of these diseases were found as cause of death or preexisting condition in patients with severe chikungunya (*2*,*13*).

**Table 1:** Expected and observed deaths during a chikungunya epidemic, Puerto Rico, July–December 2014

Cause of death (code)*	Observed deaths	Expected deaths	Difference†	Relative risk	95% CI	99% CI	p value
Acute myocardial infarction (I21–I22)	735	783	−48	0.938	0.866–1.017	0.844–1.043	0.06
All other forms of chronic ischemic heart disease (I20, I25.1–I25.9)	963	787	176	1.224	1.139–1.315	1.114–1.346	<0.01
Alzheimer's disease (G30)	1,050	1,020	30	1.030	0.962–1.102	0.942–1.126	0.20
Diabetes mellitus (E10–E14)	1,761	1,623	138	1.085	1.030–1.144	1.012–1.163	<0.01
Hypertensive heart disease (I11)	366	376	−10	0.974	0.869–1.092	0.838–1.132	0.33
Other chronic obstructive pulmonary disease (J44)	382	410	−28	0.932	0.833–1.041	0.805–1.078	0.11
Pneumonia due to other or unspecified organisms (J16, J18)	392	396	−4	0.990	0.886–1.105	0.856–1.145	0.43
Renal failure (N17–N19)	500	479	21	1.045	0.947–1.153	0.918–1.189	0.19
Septicemia (A40–A41)	456	418	38	1.091	0.984–1.210	0.952–1.250	0.05
Neoplasms (C00–D49)	2,849	2,849	0	1.000	0.960–1.042	0.947–1.055	0.50

*Causes of death were grouped according to the List of 358 Selected Causes of Death of the National Center for Health Statistics (https://ww.cdc.gov/nchs/data/dvs/Multiple_Cause_Record_Layout_2015.pdf). Codes are from the International Classification of Diseases, 10th Revision. †Observed deaths (no. cases)/expected deaths (no. cases).

**Table 2:** Number of deaths from specific causes observed during chikungunya epidemic period, Puerto Rico, July–December 2010–2015, selected according to clinical presentation of severe chikungunya deaths in Réunion Island, 2005–2006*

Cause of death (code)†							Difference between 2014 and mean of 2010–2013	Change, %
	Year							
	2010	2011	2012	2013	2014	2015		
Volume depletion, disorders of fluid, electrolyte and acid-base balance (E86–E87)	117	117	134	122	170	93	48	39
Emphysema (J43)	82	66	76	51	104	70	35	51
Conduction disorders and cardiac dysrhythmias (I44–I49)	155	146	140	154	182	142	33	22
Asthma (J45–J46)	33	45	13	24	53	37	24	84
Pneumonia (J12–J18)	419	401	339	364	402	283	21	6
Other and unspecified diseases of skin and sub-cutaneous tissue (L10–L98)	85	98	81	95	106	90	16	18
Other chronic liver disease and cirrhosis (K73–K74)	61	73	62	58	77	77	14	21
Alcoholic liver disease (K70)	45	37	49	50	58	51	13	28
Other and unspecified heart failure (I50.1–I50.9)	63	60	57	55	71	56	12	21
Diseases of pericardium and acute myocarditis (I30–I31, I40)	3	2	2	4	6	3	3	118
Meningitis and encephalitis (A83–A84, A85.2, G00, G03)	5	3	1	5	5	4	2	43
Congestive heart failure (I50.0)	229	199	207	222	204	162	−10	−5

*See reference (*2*). †Except for pneumonia, the causes of death were grouped according to the List of 358 Selected Causes of Death of the National Center for Health Statistics (https://www.cdc.gov/nchs/data/dvs/Multiple_Cause_Record_Layout_2015.pdf). Codes are from the International Classification of Diseases, 10th Revision.

## Conclusions

We found substantial excess mortality in Puerto Rico during the 2014 chikungunya epidemic, which should no longer be treated as a nonlethal disease. In addition to elderly persons, excess deaths occurred in other age groups. The main causes of death in patients with laboratory-confirmed chikungunya in hospital-based studies were similar to those in our study (*2*,*13*). 

Our study reinforces the hypothesis of the association of chikungunya with severe manifestations and deaths. Chikungunya-related death is critical to defining public health priorities, such as investment in research, vaccine development, and vector control. The evaluation of excess deaths is a tool that should be included in the assessment of chikungunya epidemics.

The results of our study are subject to several limitations. We conducted an ecologic analysis, which does not enable establishment of causality, and the investigation was based on secondary data, which may result in some inaccuracies. Therefore, some of the excess deaths we calculated might have resulted from other diseases, particularly vectorborne diseases, which have seasonal patterns of occurrence similar to those of chikungunya. However, we tried to reduce these limitations using several sources of information and accounting for other diseases that could interfere in the deaths in the region, including other viral diseases. The method used in this study is already well known and widely used to estimate deaths and hospitalizations associated with respiratory viruses and extreme weather phenomena (*14*,*15*).

Although limited, our results raise important questions about the occurrence of severe disease and increased mortality rates associated with chikungunya. Therefore, fundamental research is needed about chikungunya pathophysiologic mechanisms involving severe forms, exacerbation of preexisting conditions, and deaths. In addition to clinical studies, systematic diagnostic research of recent infection, including chikungunya, in all severe hospitalized patients during outbreaks could answer some important questions.
